# A Peptidic Thymidylate-Synthase Inhibitor Loaded on Pegylated Liposomes Enhances the Antitumour Effect of Chemotherapy Drugs in Human Ovarian Cancer Cells

**DOI:** 10.3390/ijms21124452

**Published:** 2020-06-23

**Authors:** Gaetano Marverti, Gaia Gozzi, Eleonora Maretti, Angela Lauriola, Leda Severi, Francesca Sacchetti, Lorena Losi, Salvatore Pacifico, Stefania Ferrari, Glauco Ponterini, Eliana Leo, Maria Paola Costi, Domenico D’Arca

**Affiliations:** 1 Department of Biomedical, Metabolic and Neural Sciences, Via G. Campi 287, University of Modena and Reggio Emilia, 41125 Modena, Italy; g.gozzi@holostem.com (G.G.); angela.lauriola@univr.it (A.L.); 2 Department of Life Sciences, Via G. Campi 213/d, University of Modena and Reggio Emilia, 41125 Modena, Italy; eleonora.maretti@unimore.it (E.M.); leda.severi@gmail.com (L.S.); francesca.sacchetti@alice.it (F.S.); lorena.losi@unimore.it (L.L.); sferrari591@gmail.com (S.F.); glauco.ponterini@unimore.it (G.P.); elianagrazia.leo@unimore.it (E.L.); 3 Department of Chemical and Pharmaceutical Sciences, via Fossato di Mortara 17–19, University of Ferrara, 44100 Ferrara, Italy; salvatore.pacifico@unife.it

**Keywords:** human thymidylate synthase peptidic-inhibitors, pH-sensitive PEGylated liposomes, ovarian cancer, drug-resistance, raltitrexed, 5-fluorouracil

## Abstract

There is currently no effective long-term treatment for ovarian cancer (OC) resistant to poly-chemotherapy regimens based on platinum drugs. Preclinical and clinical studies have demonstrated a strong association between development of Pt-drug resistance and increased thymidylate synthase (hTS) expression, and the consequent cross-resistance to the hTS inhibitors 5-fluorouracil (5-FU) and raltitrexed (RTX). In the present work, we propose a new tool to combat drug resistance. We propose to treat OC cell lines, both Pt-sensitive and -resistant, with dual combinations of one of the four chemotherapeutic agents that are widely used in the clinic, and the new peptide, hTS inhibitor, [D-Gln^4^]LR. This binds hTS allosterically and, unlike classical inhibitors that bind at the catalytic pocket, causes cell growth inhibition without inducing hTS overexpression. The dual drug combinations showed schedule-dependent synergistic antiproliferative and apoptotic effects. We observed that the simultaneous treatment or 24h pre-treatment of OC cells with the peptide followed by either agent produced synergistic effects even in resistant cells. Similar synergistic or antagonistic effects were obtained by delivering the peptide into OC cells either by means of a commercial delivery system (SAINT-PhD) or by pH sensitive PEGylated liposomes. Relative to non-PEGylated liposomes, the latter had been previously characterized and found to allow macrophage escape, thus increasing their chance to reach the tumour tissue. The transition from the SAINT-PhD delivery system to the engineered liposomes represents an advancement towards a more drug-like delivery system and a further step towards the use of peptides for in vivo studies. Overall, the results suggest that the association of standard drugs, such as cDDP and/or 5-FU and/or RTX, with the novel peptidic TS inhibitor encapsulated into PEGylated pH-sensitive liposomes can represent a promising strategy for fighting resistance to cDDP and anti-hTS drugs.

## 1. Introduction

Ovarian cancer is the fifth cause of mortality among women of all ages in the Western world. Although higher response rates have been achieved using the poly-chemotherapy regimens, mainly based on the combination of cyclophosphamide or paclitaxel and a platinum compound, such as cisplatin (cDDP) or carboplatin [1], many patients still die during cancer relapse and progression due to the intrinsic or acquired resistance to chemotherapy as well as the genetic flexibility of the cancer cell’s genome, resulting in multiple and often compensatory survival and proliferative signals, limiting the activity of anti-cancer strategies and compelling the continuous search for new drug combinations. 

Cisplatin is an alkylating agent that interacts with the DNA double strain structure and causes formation of adducts that prevent transcription and cellular replication and induces apoptotic cell death. In the multifactorial process of the acquired resistance to cDDP and its derivatives, the phenotypes show overexpression of DNA-repair enzymes and enzymes necessary for the synthesis of thymidine, such as thymidylate synthase (TS) and dihydrofolate reductase (DHFR), since they cause a rapid turnover of DNA [2]. As a consequence, cells resistant to cDDP are also cross-resistant to the traditional antifolates such as 5-fluorouracil (5-FU) and methotrexate (MTX) [3]. 

It is well known that tumours express higher TS levels than normal tissues and that the ovarian tissue is among those with the highest levels of TS expression, a feature that correlates with poorer patients’ overall survival [4]. 5-Fluorouracil and other antifolates, such as raltitrexed (RTX), inhibit TS, but they may cause TS upregulation in cDDP-resistant cells. The active metabolite of 5-FU, 5-fluorodeoxyuridine monophosphate (5-FdUMP), inhibits the dTMP synthesis by forming a stable ternary complex with TS and the methyl donor 5,10-methylene tetrahydrofolate (5,10-CH2THF) [5,6]. On the other hand, RTX is a TS cofactor analogue that inhibits TS with response rates similar to those of 5-FU and, differently from the pyrimidine analogues, is not incorporated into DNA. It has been licensed in many countries for the treatment of metastatic colorectal cancer [7,8]. Preclinical and clinical studies have demonstrated a strong association between development of resistance to both 5-FU and RTX and increased TS expression [9,10,11]. 

Exposure of cancer cells to 5-FU or other antifolate TS inhibitors acutely upregulates TS, probably due to the inhibition of a negative-feedback mechanism in which the TS protein binds its own mRNA and inhibits its translation [7,12,13]. In addition, TS behaves as an oncogene [14] by interfering with the expression of proteins involved in the regulation of proliferative and survival pathways such as c-myc and p53 by binding their mRNAs [15,16]. However, expression of wild-type p53 has been shown to be required for 5-FU- and RTX-induced antitumor effects [17,18]. Therefore, new strategies helpful to overcoming these TS-focused molecular mechanisms of chemo-resistance are required.

As one such strategy to obtain tumour cell death without inducing TS overexpression, we designed oligopeptides able to modulate the equilibrium between the active TS conformation and the inactive one. The novel mechanism of action involves binding at the monomer-monomer interface of the enzyme, rather than at the catalytic pocket, with stabilization of the inactive conformation and decrease of the abundance of the active form of the protein. Among these, the LR (LSCQLYQR) peptide inhibited the growth of ovarian carcinomas (OCs) following transfection by means of a commercial peptide delivery system, SAINT-PhD, while it left the TS cellular levels essentially unchanged [19,20]. More recently, among the peptides developed by modifying the LR lead to improve TS inhibition and the anticancer effect, the D-glutamine-modified peptide at position 4 ([D-Gln^4^]LR) displayed the best growth inhibition of both cDDP-sensitive and -resistant OC cell lines [21]; it was more active than LR and 5-FU and affected the TS/DHFR expression pattern similarly to LR. 

Proteomic studies have shown that, in human OC cell lines, these peptides modulate the expression of a panel of six proteins, including TS and other important folate-related enzymes such as phosphoribosylglycinamide formyltransferase (GART) and serine hydroxymethyltransferase (SHMT1) [22]. The modulation of these proteins was markedly different from that induced by pemetrexed (PMX), another TS inhibitor and a 5,10-CH2THF analogue, which, again, unlike the peptide, binds at the TS active site.

The possibility of combining conventional cytotoxic drugs with new agents that specifically interfere with key pathways controlling cancer cell survival and proliferation is considered an interesting and promising therapeutic approach. Indeed, if the cellular targets for these new agents and/or their mechanism of action are different from those of conventional cytotoxic drugs, and if the effects of the drugs combined on key pathways are concordant, their combination may act on the sensitivity of cancer cells synergistically. 

Recently, the hydrophilic peptide LR has been delivered into OC cells by exploiting such biocompatible and efficient tools as solid lipid nanoparticles (SLNs) [23] and pH sensitive liposomes [24]. The ability to inhibit the cDDP-resistant OC cell growth demonstrated the efficacy of these nanosystems in the internalization of the peptide into cells while preserving its activity. Enhanced effects are expected when employing PEGylated liposomes. In fact, PEGylation represents a very useful method for achieving long circulation time for liposomes, hence allowing them more time for targeting tumours via the enhanced permeability and retention (EPR) effect [25]. The PEGylated pH-sensitive liposomes were characterized and compared favourably with non-PEGylated ones regarding surface hydrophilicity, stability in serum, interaction with macrophages, drug encapsulation efficiency and drug release [26]. Also, the well-characterized intracellular mechanism of action of the [D-Gln^4^]LR peptide delivered using the SAINT-PhD delivery system was unaltered when the latter was replaced by these liposomes. While the commercial delivery system proved useful in the early cellular studies, the PEGylated liposomes strategy is deemed necessary to implement further in vivo studies of the [D-Gln^4^]LR peptide. 

In the present study, we test the efficacy of combinations of RTX, cDDP and 5-FU with the ([D-Gln^4^]LR octapeptide, delivered into OC cells by either the SAINT-PhD delivery system or by PEGylated pH-sensitive liposomes. More specifically, we investigate their cytotoxicities and abilities to perturbate the cell cycle phase distribution. The microtubule polymer stabilizer paclitaxel was also included in the described combination list, because the drug is largely used in ovarian cancer chemotherapy and shows a different mechanism of action. To this aim, three different tumour-cell treatment schedules were tested. Special attention was focused on the synergistic effects exhibited by the combined cell treatments and the role of sequentially thereon.

## 2. Results

### 2.1. Sequence-Dependent Synergistic Antiproliferative Effects of [DGln^4^]LR/Drug Combinations

In cancer cells treated with 5-FU or RTX, alterations in TS expression were associated with drug resistance response [7,12,13,14]. Despite the fact that these compounds and peptides share the same target (TS), they have different molecular mechanisms of action and, thus, are not a priori reciprocally competitive. Therefore, we investigated the potential cooperative antitumor effect of the [DGln^4^]LR peptide in combination with these drugs, as well as with cDDP and paclitaxel, two other chemotherapeutic agents used for the treatment of OC patients. We used cDDP-sensitive A2780 and 2008 cell lines and their cDDP-resistant counterparts, A2780/CP and C13*, to evaluate the combined effect on cell proliferation of [DGln^4^]LR with either 5-FU, RTX, cDDP or paclitaxel by the synergism Quotient (SQ) analysis. Among the different cell lines examined the IC_50_ values for the drugs alone varied depending on the resistance phenotype (Table S1). 

**Figure 1:**
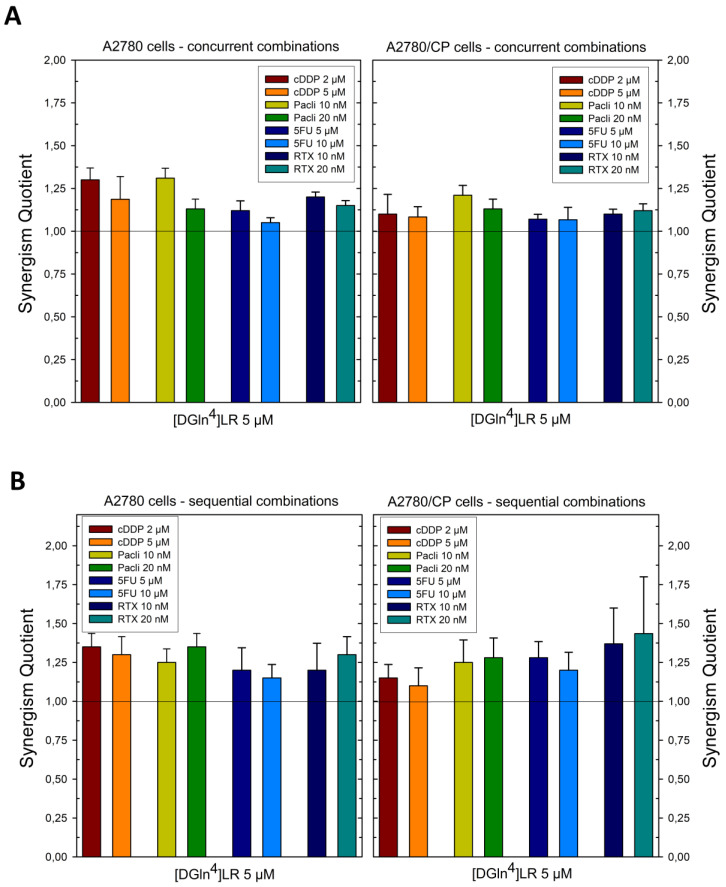
Effects of scheduled combinations of the [D-Gln4]LR peptide with cDDP, paclitaxel, 5FU and RTX on the SQ values in A2780 and A2780/CP cell lines. (**A**) concurrent combinations for 72 h. (**B**) Sequential combinations, as described in Section 4. The bars represent the mean of duplicate cell counts on three separate experiments and indicate the result of the inhibition of drug combination divided by the sum of the inhibition of a single drug to obtain the values of SQ. Error bars, SD.

The [D-Gln^4^]LR peptide was delivered by the SAINT-PhD system to A2780 and A2780/CP cells either concurrently or sequentially with the four drugs, each administered at concentrations lower than the IC_50_ value (IC30) obtained with the drug alone on the tested cell line. As shown in the diagrams in Figure 1, and synthesized by the heat map in Figure 4, most of the results of the concurrent administration (sequence I) demonstrate additive or moderately supra-additive effects, as indicated by SQ values around or slightly higher than 1, even in the cDDP-resistant A2780/CP cell line. On the other hand, administration of the peptide followed by the drug (sequence II) caused an overall increase in cell killing. This was evident with both cell lines in the treatment with the lower doses of all four drugs, while, at the higher doses, only 5-FU showed an improved performance with sequence II over the concurrent administration. 

**Figure 2:**
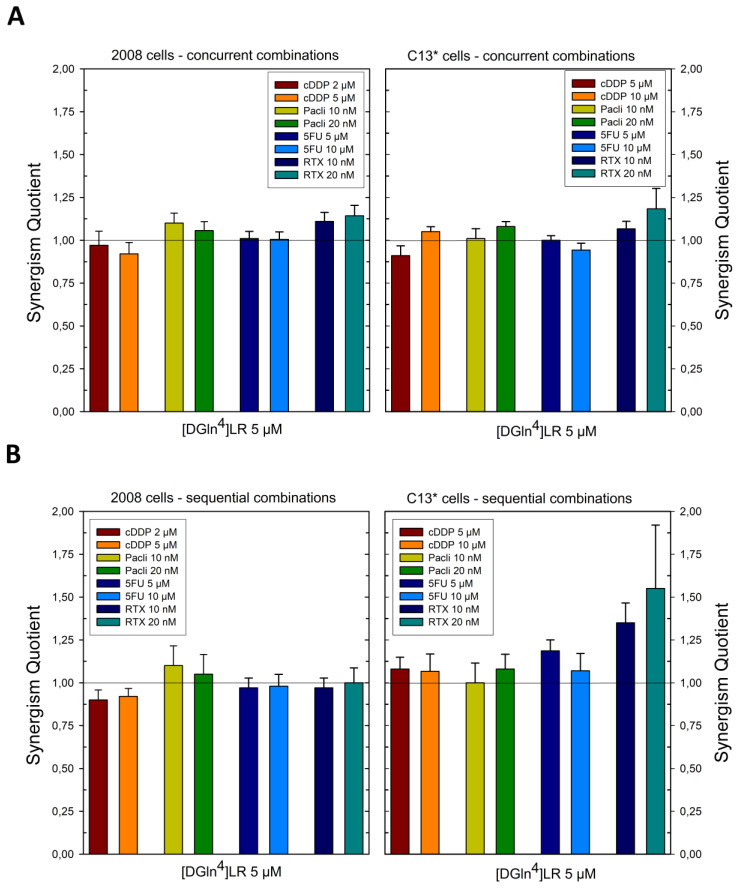
Effects of scheduled combinations of [DGln4]LR peptide with cDDP, paclitaxel, 5FU and RTX on the SQ values in 2008 and C13* cell lines. (**A**) concurrent combinations for 72 h. (**B**) Sequential combinations as described in Section 4. The bars represent the mean of duplicate cell counts on three separate experiments and indicate the results of the inhibition of drug combinations divided by the sum of the inhibition of a single drug to obtain the values of SQ. Error bars, SD.

**Figure 3:**
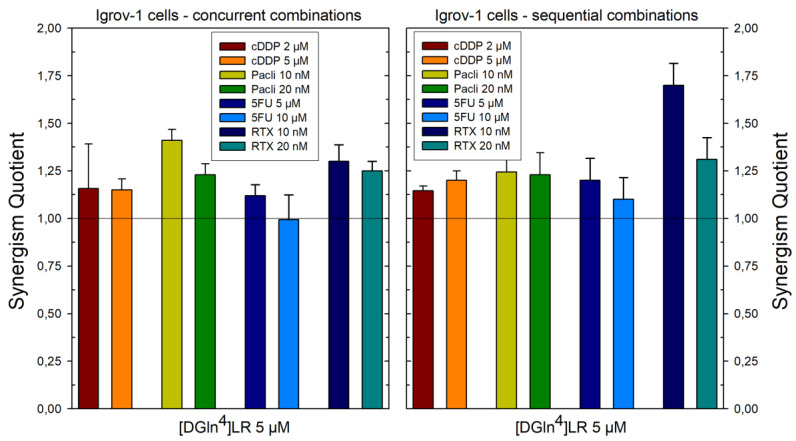
Effects of scheduled combinations of [DGln4]LR peptide with cDDP, paclitaxel, 5FU and RTX on the SQ values in IGROV-1 cell line. (Left panel) Concurrent combinations for 72 hr. (Right panel) Sequential combinations as described in Section 4. The bars represent the mean of duplicate cell counts on three separate experiments and indicate the results of the inhibition of drug combinations divided by the sum of the inhibition of a single drug to obtain the values of SQ. Error bars, SD.

**Figure 4:**
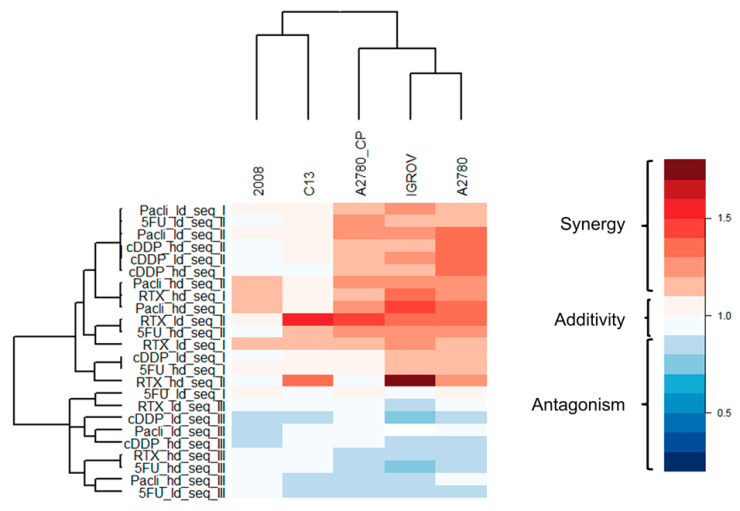
Heatmap of the synergism quotient values of the tested combinations (rows) against the different cell lines (columns). Colour code: red, SQ > 1; blue, SQ < 1. The reported dendrogram was built based on the dissimilarity matrix using Euclidean distances and the complete linkage method. ld: lower dose; hd: higher dose; Pacli: paclitaxel, Seq I, II, III: combination sequences I, II and III as described in the text.

With the second pair of cell lines, 2008 and C13*, concurrent administration was additive for cDDP, 5-FU, and paclitaxel at the lower dose, supra-additive at the higher dose of paclitaxel and for RTX at both doses (Figure 2 and Figure 4). Sequential exposure to [D-Gln^4^]LR and then to the drugs did not enhance cell killing with the cDDP-sensitive 2008 cells with respect to the concurrent administration. On the contrary, with the resistant C13* cells both 5-FU and RTX increased their cytotoxicities when administered after [D-Gln^4^]LR; in particular, RTX exhibited a synergistic efficacy at the lower dose. A general enhancement of cytotoxicity, with SQ values higher than unity, was obtained with both combined treatments of IGROV-1 cells, at almost all drug doses employed (Figure 3 and Figure 4). Advantages of the sequential treatment according to schedule II over the concurrent treatments were demonstrated by supra-additive or synergistic SQ values obtained with 5-FU and RTX using this schedule, in particular at the lower concentrations tested. Interestingly, this scheduled combination with RTX 10 nM reached a synergistic SQ value of about 1.7. On the other hand, when drug treatment preceded exposure to the peptide (sequence III) the anti-proliferative results were antagonistic with all drugs and cell lines tested (Figure 4). As summarized in Figure 4, the IGROV-1 and A2780 cell lines show similar overall response profiles to the tested combinations. In fact, almost all the combinations, except 5-FU at low dose administered after [D-Gln^4^]LR peptide, showed synergistic activity. On the other hand, all the combinations in which the [D-Gln^4^]LR peptide was administered after the drugs showed antagonism or, in a few cases, additive activities. Regarding the tested combinations: (i) RTX-high dose in concurrent administration with the [D-Gln^4^]LR peptide has a synergistic activity towards all the tested cell lines; (ii) low-dose RTX and high-dose 5-FU administered after the [D-Gln^4^]LR peptide show synergistic activity towards all cell lines, except the 2008 cells; (iii) high-dose RTX and paclitaxel in the concurrent administration and high-dose paclitaxel administered after the [D-Gln^4^]LR peptide showed synergistic activity towards all cell lines, except C13*.

Taken together these results show that scheduling may be crucial for potentiating the antitumor effect of [D-Gln^4^]LR. In particular, its combination with 5-FU and RTX, i.e., drugs that target folate cycle enzymes, but also with cDDP and paclitaxel, shows enhanced effectiveness when the drugs are administered after the peptide.

### 2.2. [DGln^4^]LR Combination with Chemotherapy Drugs Cause Great Perturbation of Cell Cycle and Promotes Apoptosis

**Figure 5:**
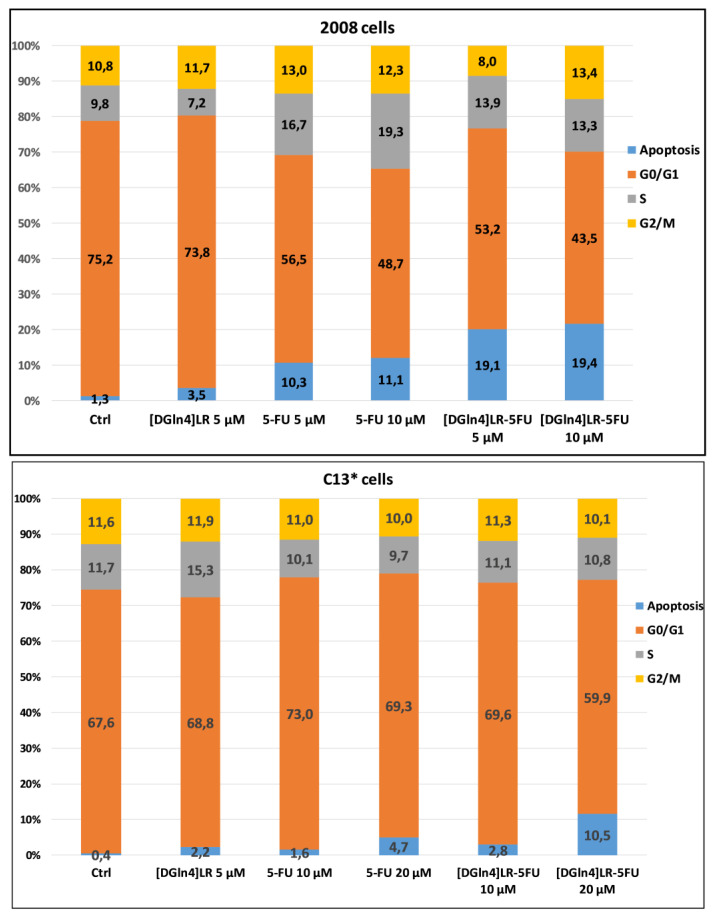
Effect of the [DGln4]LR peptide and 5-FU alone and in combination on the cell cycle phase distribution of 2008 and C13* cells by cytofluorimetric analysis of the DNA content by PI staining. After a 72 exposure to 5 µM [DGln4]LR and to the indicated concentrations of 5-FU alone or in concurrent combinations. Cells were processed according to methods described in Section 4. The inserted numbers indicate the percentages of cells in the different phases of the cell cycle and are the mean of two/three experiments. The error bars are omitted for a clearer visualization.

**Figure 6:**
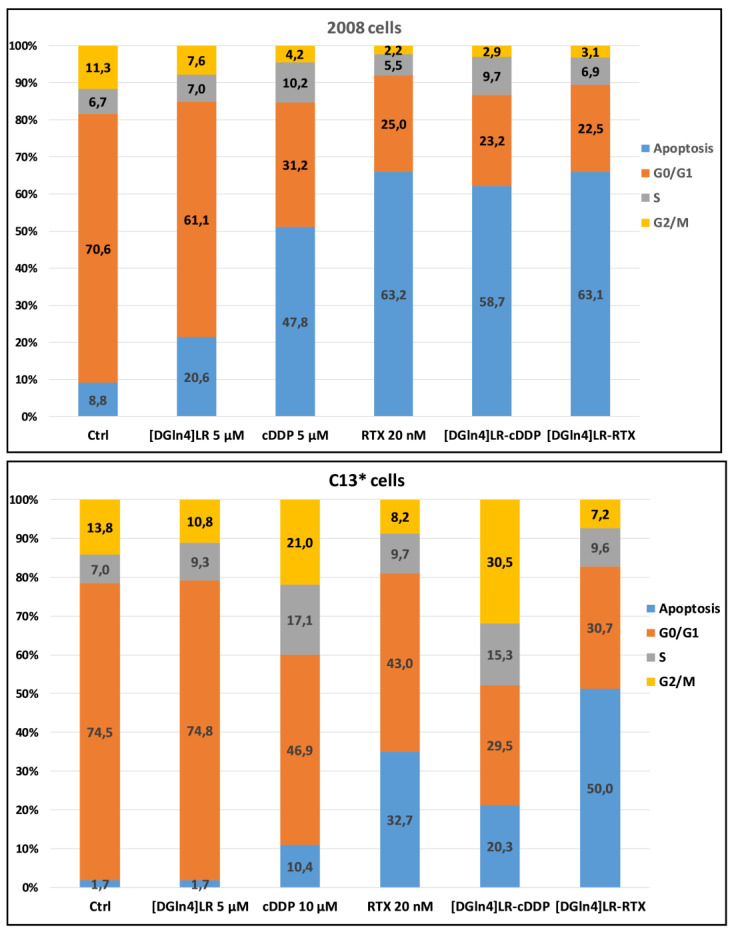
Effect of the [DGln4]LR peptide and cDDP alone and in combination on the cell cycle phase distribution of 2008 and C13* cells by cytofluorimetric analysis of the DNA content by PI staining. After a 72 exposure to 5 µM [DGln4]LR and 5 µM (2008 cells) or 10 µM (C13* cells) cDDP or 20 nM RTX alone and in concurrent combinations, cells were processed according to Section 4. The inserted numbers indicate the percentages of cells in the different phases of the cell cycle and are the mean of two/three experiments. The error bars were omitted for a clearer visualization.

Looking for a possible mechanism underlying the antiproliferative activity and correlating with the additive or synergistic effects of the combination between [DGln^4^]LR with the chemotherapy drugs cDDP, 5-FU and RTX, a perturbation of the cell distribution in the different phases of the cell cycle was tested in cytofluorimetric experiments (DNA content analysis) on the 2008 and C13* cell lines. The percentage of cells in the different phases of the cell cycle is reported in Figure 5 and Figure 6, Table S2 and Figures S1–S3. 

Figure 5 and Table S2 report the results obtained with 2008 and C13* cell lines treated with 5 µM peptide and two pairs of 5-FU concentrations (5–10 µM and 10–20 µM), in sensitive and resistant cells, respectively. After 72 h, untreated cells of both lines showed a normal diploid distribution presenting fast proliferation characteristics. 5-FU, both alone and in combination, had a very scant effect in perturbing the distribution of the C13*-resistant cells in the different phases of cell cycle, even if a synergistic but modest accumulation of cells in sub-G1 phase was observed with 20 µM 5-FU, a finding indicative of apoptotic cell death (hypodiploid cells). 

On the contrary, 2008 cells were more sensitive to both concentrations of 5-FU (5–10 µM) showing a reduction of cell accumulation in the G0/G1 phase, coupled with an increase of cell accumulation in both S and sub-G1 phases. The increase of sub-G1 phases (apoptotic cells) was almost doubled by combination with the peptide, reaching approximately 20% of synergistic cell killing (SQ = 1.38-1.33).

The other two chemotherapy drugs, cDDP and RTX caused a great decrease of sensitive cell accumulation in the G0/G1 phase that paralleled with a remarkable increase of percentage of the sub-G1 phase cell population (apoptotic cells), The combination of the peptide with each chemotherapy drug produced a synergistic accumulation of hypodiploid 2008 cells in comparison to single drug treatment (SQ = 1.1-1.2). This effect was also accompanied by a decrease of cell accumulation in all phases of cell cycle (Figure 6, Figures S1 and S2, Table S3). Notably, treatment with each drug alone deranged the cell-cycle phase distribution even in the resistant C13* cells, in which the percentage of the sub-G1 phase population was synergistically increased by combining the peptide with cDDP (SQ = 1.7) and with RTX (SQ = 1.13). RTX alone was also very effective as an apoptosis-inducing agent, particularly in sensitive cells.

Interestingly, an additive and a synergistic accumulation of cell population in the sub-G1 phase was also observed in the sensitive and resistant cells, respectively, already after 48 hr treatment with combinations of the peptide with either cDDP or RTX (Table S3 and Figure S3).

### 2.3. Liposome Characterization

Next, we move on, and pass from the SAINT-PhD delivery system to the engineered PEGylated liposomes, which represent an advancement towards a more drug-like delivery system. The PEGylated liposomes strategy is deemed necessary to implement further in vivo studies of the [D-Gln^4^]LR peptide in combination with standard drugs. 

Liposomes showed a size of about 200 nm with a good dimensional homogeneity, as shown by the low polydispersity index (PDI) value (Table 1). Moreover, the PDI did not show any significant alteration after drug addition, indicating that neither the homogeneity nor the stability of the liposomes were affected by the inclusion of the drug. The same occurred with the surface charge. 

**Figure 7:**
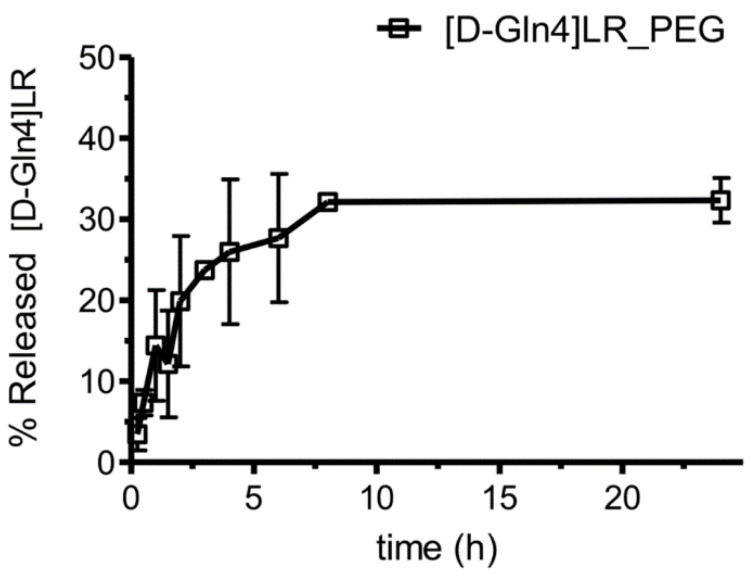
Time course of the [D-Gln4]LR release percentage from [D-Gln4]LR-PEGylated liposomes in phosphate buffer saline (PBS; pH 7.4). Error bars, SD; where not visible, error bars did not exceed symbol size.

The peptide was efficiently encapsulated in the liposomes. The drug loading (DL) was 21.03 ± 1.66 µg peptide/mg of lipid and the encapsulation efficiency approximately 40%. Consistently with the two-fold drug/lipid ratio used in the preparation, this formulation exhibited a DL twice that of the already reported homologue formulation [26]. The obtained loaded liposomes efficiently retained the peptide: after an 8 h incubation, only 30% of the initially encapsulated peptide was released and no further escape was observed in the next 16 h (Figure 7).

### 2.4. Cytotoxicity of Peptide-Loaded Liposomes

The antiproliferative activity of the peptide encapsulated in these optimized liposomes (PpHL) was tested by the MTT assay on three OC cell lines, the cDDP-resistant C13* cells and their sensitive counterparts, the 2008, and the IGROV-1 cell lines, the latter sensitive to cDDP too. 

**Figure 8:**
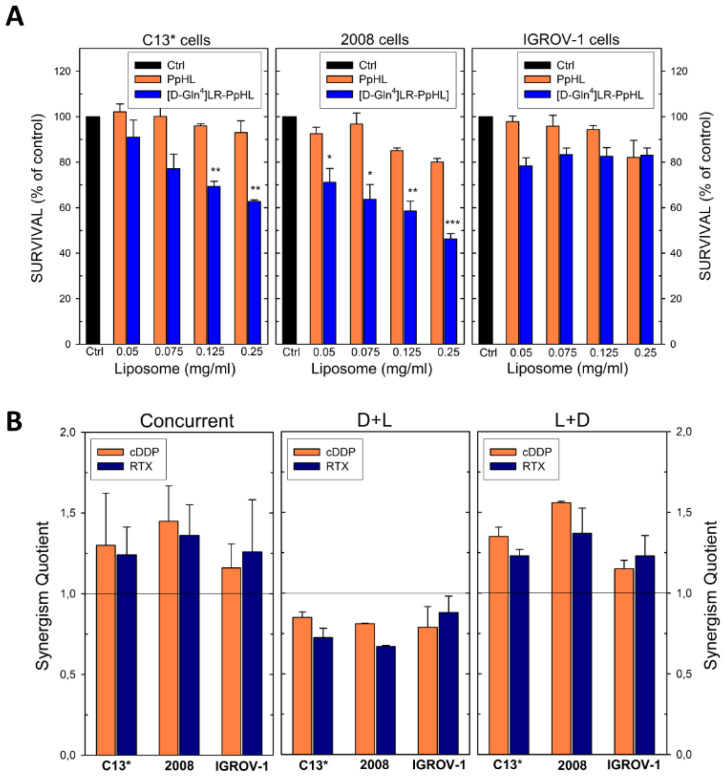
MTT test on C13*, 2008 and IGROV-1 cell lines. Cells were incubated with increasing amounts of [D-Gln4]LR-PpHL and PpHL (**A**) for 15 h, followed by a further 48-h incubation after the removal of the treatment. The results were expressed as percentage of cell growth with respect to the control (untreated cells), set at 100% of viability. Error bars, SD. Comparison between groups was performed by the ANOVA one-way test. Statistical significance levels were defined as * *p* < 0.05, ** *p* < 0.01 and *** *p* < 0.005. (**B**) The synergism of cell growth inhibition is reported as synergism quotient (SQ). The Concurrent chart corresponds to simultaneous liposomes + drug-exposure; D+L chart corresponds to sequential exposure in which the drugs (cDDP or RTX) was given 24 h before liposomes; L+D chart corresponds to the reversed sequential exposure. Error bars, SD.

In the first experiment, the cells were exposed to increasing concentrations of the peptide-loaded liposomes ([D-Gln^4^]LR-PpHL) according to the optimised protocol reported, using the unloaded carriers as a control. Moreover, the free [D-Gln^4^]LR peptide at the equivalent concentration was used for comparison. The elaborated results are reported in Figure 8. The unloaded liposomes (PpHL) were not toxic towards all cell lines since cell viabilities higher than 85% were observed for all the liposome concentrations and cells considered (Figure 8A).

Concerning the effect of [D-Gln^4^]LR-PpHL, a marked difference in cytotoxicity between loaded and unloaded carriers was observed, particularly with the cDDP-sensitive 2008 cell line. Indeed, at the higher liposome concentration, 0.25 mg/mL, a 50% viability was obtained with these cells, while a slightly higher survival, 63%, was exhibited by the cDDP-resistant C13* cells. On the other hand, IGROV-1 cells, despite their cDDP-sensitivity, proved quite resistant to the peptide-loaded liposomes, exhibiting cell viabilities about 80% with all the amounts of liposomes employed. It should be noticed that IGROV1 cells are known to exhibit a different behaviour to drug treatment. Despite being sensitive to cisplatin in vitro, they are resistant to Asta Z, and present an intermediate drug response to adriamycin. Finally, concerning the effect of drug loading, its doubling caused only a moderate increase in cytotoxicity on the C13* cells, with a 63% survival vs a 70% survival measured with the original preparation [26], a finding likely due to the saturation of the intracellular target enzyme, hTS. 

The importance of these liposomes as vehicles for the peptide internalization into cells was confirmed by the inability of the free [D-Gln^4^]LR peptide to interfere with the growth of all three cell lines [26]. 

### 2.5. Sequence-Dependent Synergistic Antiproliferative Effect of Peptide-Loaded Liposomes in Combination with RTX or cDDP

The peptide-loaded liposomes [D-Gln^4^]LR-PpHL at a concentration of 0.125 mg/mL, corresponding to 2.12 µM overall extracellular peptide concentration, was combined with RTX and cDDP at different concentrations, according to the cell line sensitivities to these drugs, 10 nM RTX and 5 μM cDDP for C13*, 10 nM RTX and 2.5 μM cDDP for IGROV-1 and 2008 cells, respectively. Peptide-loaded liposomes combined with the two anticancer drugs showed greater efficacy against both cDDP-sensitive and -resistant cell lines when administered concurrently or sequentially (liposome, L+drug, D) (sequences I and II, respectively), while the reversed schedule (D+L, sequence III) produced an antagonistic effect; the combination sequences leading to the synergistic antiproliferative effect are the same observed with the SAINT-PhD delivery system (Figure 1, Figure 2, Figure 3 and Figure 4). Noteworthy, sequences I and II synergistically killed even IGROV-1 cells, i.e., the least responsive to the peptide-loaded liposomes alone (Figure 8A). The SQ values obtained are shown in Figure 8B.

## 3. Discussion

The [D-Gln4]LR peptide and its lead, LR, have exhibited cancer cell-growth inhibitory activity by mainly reducing the abundance of the active form of hTS, and, unlike 5-FU and PMX, without inducing overexpression of the enzyme [19,21], but even by down-modulating the expression of other folate pathway genes, DHFR and AICAR transformylase (ATIC) [22]. Cells that acquire resistance to classical TS inhibitors because of an enhanced TS expression exhibit general cross-resistance with platinum-based drugs [3] and display cross-resistance to antifolates such as RTX [27,28]. 

Antifolates targeting hTS are not well known in OC therapy. All those inhibitors bind at the protein active site and this cause the loss of the translational control and TS levels regulations [29]. Our hypothesis was that if TS levels are reduced, drug resistance mechanisms will be limited or prevented. So, we propose a change of paradigm in TS inhibition based on new drugs that, unlike the well-known, traditional TS inhibitors (RTX, PMX, 5FU), bind at the protein interface. [22,30,31,32]. These compounds can be combined with platinum drugs, RTX, PMX or 5FU to keep TS levels low and contrast drug resistance development.

Owing to the intricate biochemical interconnections and the difference in biological pathway modulation between the peptides and the classical drugs that, in order to explore strategies to overcome tumour drug resistance and achieve greater therapeutic gains, the present in vitro study has investigated the effects of combining the novel TS inhibitor peptides with cDDP [1], as well as some classical antifolate agents. To provide a mechanistic interpretation of the results of our combination experiments, we first start from known individual drug effects as well as some drug-combination literature reports.

Combination of cDDP with classical folate cycle inhibitors, such as 5-FU, has been explored in experimental and clinical studies and has exhibited a pronounced activity against various types of human tumours, including chemo-resistant ovarian carcinoma [33,34]. However, the response and toxicity varied considerably according to the schedule and dose used [35,36]. The explanation of the synergistic response attained with 5-FU plus cDDP likely involves more than one mechanism. On one hand, it was correlated to increased TS inhibition related to higher levels of FdUMP, the active 5-FU metabolite. This, in turn, was associated with an increase in the intracellular reduced folate levels in the tumour cells by inhibition of the transport of L-methionine by cDDP, a well-known biochemical mechanism by which cDDP potentiates the cytotoxicity of 5-FU, [33,37]. However, a greater degree of fragmentation of both nascent and parent DNA was indicated as another possible mechanism of the 5-FU/cDDP synergism [38]. Finally, the better objective response to this drug combination exhibited by 5-FU-resistant patients has been explained by showing that TS inhibition by treatment with platinum drugs and 5-FU also occurs at the transcriptional level [39]. Overall, these explanations establish a causal relationship between hTS inhibition and a decreased efficiency of the mechanisms of repair and substitution of damaged DNA. This might apply to our case as well: the peptide delivered by the liposomes, before (sequence II) or concurrent (sequence I) with cDDP, causing the folate cycle enzyme inhibition, may hamper the repair and substitution of the DNA damaged by cDDP. Additional support to this mechanistic hypothesis comes from the previously reported evidence that treatment of OC cells with the TS inhibiting peptide [D-Gln^4^]LR induces deregulation of such folate-related proteins as TS, DHFR and SHMT1 [21]. In particular, the idea that cDDP and the peptide may act synergistically on the folate pathway is reasonable since TS and DHFR operate in the same metabolic cycle [40,41,42].

Other nodal proteins of the folate pathway were found to be affected by the LR and [D-Gln^4^]LR peptides. Among these, ATIC, is an enzyme involved in purine biosynthesis, and NME2, also known as nucleoside diphosphate kinase B, plays a major role in the synthesis of nucleoside triphosphates other than ATP [22]. Because both ATIC and NME2 are down-regulated following treatment with our peptidic inhibitors and are modulated at the gene level by c-Myc [43], a role of this proto-oncogene in the mechanism of action of the two peptides seems likely. Furthermore, the involvement of c-Myc in the mechanism of the observed synergistic effect of the peptides with cDDP and 5-FU is suggested by the known important role played by the downregulation of c-Myc in the cell response to cisplatin and to 5-FU [43,44,45].

The observed synergy of the peptide with RTX might be related to several different effects: RTX and/or its polyglutamated derivatives reduces the purine biosynthesis as suggested by the increase in the intracellular levels of phosphoribosylpyrophosphate (PRPP) following a 24 h exposure of colon carcinoma cells to RTX [46]. Because RTX is also a moderate direct inhibitor of DHFR, the observed peptide/RTX synergy could result from inhibition of this enzyme and the consequent increase of PRPP levels in the cell via inhibition of purinic biosynthesis [45], thereby potentiating the cellular inhibitory effects of the peptide. 

In addition, the synergistic effect of combining the peptides with cDDP or the other drugs may derive from mis-incorporated genomic uracil, resulting from TS inhibition, which induces DNA damage and early cell-cycle arrest as a result of BER activity, and is a critical determinant of sensitivity to antifolate-based TS inhibition [46].

Our findings on the dependence of the effects of the combination of peptides and RTX on the administration schedule correlate with previous reports where synergistic effects, were obtained by a sequential exposure to the DHFR inhibitor methotrexate (MTX) followed by RTX [47]. Actually, the interaction between different antifolates or between antifolates and a Pt drug quite generally follows a preferential treatment schedule. In most cases, simultaneous and continuous administration of RTX and cisplatin, or the sequential administration of RTX followed by cisplatin, produce the highest cytotoxicity, while the reversed sequential administration produces antagonistic effects and thus resulting inappropriate [42].

In our case, the observed sequence-dependent synergy might be accounted for in connection with the finding that HSP90 and TRAP1, that are critical regulators of survival of tumour cells, are among the proteins down-regulated by the peptides. TRAP1 has been described as a mitochondrial chaperone of HSP90AA1. Effective cytoprotection may require TRAP-1 phosphorylation by the mitochondrial-localized kinase, PINK1, which associates with TRAP-1 in vivo. The high expression of TRAP-1 in cancer has been implicated in the inhibition of mitochondrial apoptosis, suppression of ROS production and acquisition of resistance to standard chemotherapeutics [48]. On the opposite hand, decreased TRAP1 expression leads to the accumulation of ubiquitinated/misfolded proteins and proteotoxic stress, a condition that makes cells more sensitive to apoptotic insults, including those caused by treatment with such drugs as cDDP [1]. HSP90 is a ubiquitously expressed molecular chaperone that is involved in the posttranslational folding and stability of multiple mutated, chimeric and over-expressed signalling proteins that promote the growth and/or survival of cancer cells [49]. Previous studies have shown that HSP90 is highly expressed in OC, and a sub-member, HSP90AA1, has been shown to be required for the survival and proliferation of human OC SKOV3 cells. High levels of HSP90AA1 can increase chemo-resistance to cisplatin of SKOV3 cells [50]. Moreover, the concomitant overexpression of ATIC and TRAP1 has been associated to chemoresistance to both cisplatin and 5-FU [51,52]. Therefore, inhibition of HSP90 could potentially act as a promising adjuvant to chemotherapies to overcome drug resistance. Inhibition of these two chaperones, HSP90AA1 and TRAP1 has been already reported following treatment with the antifolate drug MTX and yielded a synergistic effect by interfering with the synthesis of purines [53]. In this light, disabling of HSP90 cancer networks in their multiple subcellular compartments, mitochondrial and cytosolic, by our peptidic inhibitors, may cause irreversible collapse of mitochondria, degradation of HSP90 client proteins in the cytosol, and tumour cell killing by apoptosis and/or autophagy, thus favoring the action of both cDDP and RTX. Also, because RTX induces mitochondrial mediated apoptosis in SGC7901 human cancer cells by disrupting the PI3K/AKT/Hsp-90 cascade and mitochondrial integrity [54,55,56], a peptide-induced HSP90 disabling may explain the higher efficacy of a sequential treatment schedule.

The combination synergy of the peptide with paclitaxel, that like the other mentioned chemotherapeutic drugs, is frequently used in OC therapy, is justified by several experiments that report, already at the clinical level, on the positive effects of the combination of this microtubule stabilizer with folate-cycle inhibitors and/or platinum drugs [57,58,59]. 

## 4. Materials and Methods 

### 4.1. Materials

Raltitrexed (RTX) and paclitaxel were purchased from Selleckchem (Houston, TX, USA) and were dissolved in DMSO immediately before addition to the cell cultures. Cisplatin (cDDP) was obtained from Santa Cruz Biotechnology. All other chemicals were purchased from Sigma–Aldrich S.r.L. (Milan, Italy), except otherwise indicated. 

Reagents for liposome formulation: cholesteryl hemisuccinate (CHEMS) purchased from Sigma–Aldrich (St. Louis, MO, USA); 1,2-dioleoyl-sn-glycero-3-phosphoethanolamine (DOPE) and [N-(Cabonyl-methoxypolyethyleneglycol-2000)]-1,2-distearoyl-sn-glycero-3-phosphoethanolamine sodium salt (DSPE_PEG) purchased from Lipoid (Ludwigshafen, Germany). The active peptide [D-Gln^4^]LR and the internal standard (IS) for quantitative analysis, LR-Ala7, were synthesized as previously described [21,22]. All the other reagents were of analytical grade.

### 4.2. Cell Lines

The five human cancer cell lines of ovarian origin, IGROV-1, A2780, A2780/CP, 2008 and C13*, were grown as monolayers in RPMI 1640 medium containing 10% heat-inactivated foetal bovine serum and 50 µg/mL gentamycin sulphate. The 2008 cell line was established from a patient with serous cystadenocarcinoma of the ovary, and their cisplatin (cDDP)-resistant variant C13* cell line, was developed by monthly exposure to cDDP, followed by chronic exposure to stepwise increases in cDDP concentration [60,61]. The human ovarian carcinoma A2780/CP cells are about 10 fold resistant to cDDP and derived from the parent A2780 cell line [62]. The IGROV-1 cell line, originating from an ovarian carcinoma of a 47 years old woman, was established in monolayer tissue culture. The IGROV-1 cell line exhibits an epithelial character, highly tumorigenic properties and a low doubling time. In addition, there are consistent cytogenetic markers in which oncogenic rearrangements can occur. IGROV-1 is therefore proposed as a model for experimental studies of human ovarian adenocarcinoma, including molecular and cell biology, preclinical pharmacology and experimental therapeutics [63]. The cells were incubated at 37 °C under 5% CO_2_ for at least 24 h before treatment with the peptides. All cell media and serum were purchased from Lonza (Lonza Group Ltd., Basel, Switzerland). Cultures were equilibrated with humidified 5% CO_2_ in air at 37 °C. All studies were performed in Mycoplasma negative cells, as routinely determined with the MycoAlert Mycoplasma detection kit (Lonza Group Ltd., Basel, Switzerland). 

### 4.3. Cell Transfection with [D-Gln^4^]LR Peptide by Means of the Delivery System SAINT-PhD

Treatment with peptide was performed according to the standard transfection protocol of the SAINT-PhD delivery system (Synvolux Therapeutics, Groningen, the Netherland). Complexes of peptide (µg) with SAINT-PhD (µL) were prepared at a ratio of 8 µg/20 µL (which corresponds to a concentration of 5 µM final). For each protein sample, complexes were prepared as follows: for the treatment of a 24-well, an appropriate amount of the peptide was diluted in HBS, then SAINT-PhD was pipetted into the solution without vortexing. The mixture was incubated for 5 min at room temperature, then filled with serum-free medium. The culture medium was aspirated from the cells, the SAINT-PhD/peptide complex was added to the wells and incubated 4 h (37 °C; 5% CO_2_). After this time, complete RPMI was added to reach the appropriate volume.

### 4.4. Liposome Characterization

DOPE:CHEMS:DSPE_PEG (PpHL) liposomes were prepared using the Reverse Phase Evaporation technique followed by homogenization using Ultraturrax (Ika-euroturrax T 25 basic, IkaLabortechnik, Staufen, Germany). Briefly, phospholipid solutions in chloroform at fixed concentration, DOPE:CHEMS:DSPE (22.8: 15.2: 2 mM) for nPpHL liposomes and DOPE:CHEMS:DSPE_PEG (22.8: 15.2: 2 mM) for PpHL liposomes, were employed and an amount of 4 mg of [D-Gln^4^]LR was loaded in the liposomes obtaining a final suspension of 15 mg/mL in HBS (Hepes Buffer Saline with 20 mM Hepes and 135 mM NaCl) [29]. Size, size homogeneity and surface charge of the liposomes diluted in water (1:5 v:v) were measured by Zetasizer Nano ZS analyser system (Zetasizer version 6.12; Malvern Instruments, Worcs, UK), equipped with a 4 mW He–Ne laser (633 nm) and a DTS software (Version 5.0). Measurements were performed in triplicate and each measurement was averaged over at least 12 runs.

Drug loading (DL), encapsulation efficiency (EE) and drug release were analysed by liquid chromatography (Agilent 1200 Series LC system; Agilent, Technologies, Milan, Italy) coupled with triple-quadrupole mass spectrometry (LC-MS/MS) (Agilent 6410 triple quadrupole-mass spectrometer, Agilent technologies, Milan, Italy), as described previously [26].

### 4.5. Cytotoxicity of Liposome-Drug Combinations by MTT Assay

The cytotoxicity assay was performed by the (3-(4,5-dimethylthiazol-2-yl)-2,5-diphenyl tetrazolium bromide; Sigma, Milan, Italy) assay (MTT assay) [64]. Briefly, C13*, 2008 and IGROV-1 cell lines were seeded at a density of 30,000 cells/well in 24-well plate in complete RPMI-1640 medium for at least 24 h. Immediately prior to the cell treatment, the culture medium was aspirated from each well and replaced with 500 μL of complete medium, containing either the unloaded or the [D-Gln^4^]LR-loaded liposomes at concentrations of 0.05, 0.075, 0.125 and 0.250 mg/mL; moreover, cells were treated, for comparison, with equivalent amounts of naked peptide. Cells were then incubated in complete medium suspension in 5% CO_2_ incubator at 37 °C for 15 h. Then, they were washed with PBS, added with 500 μL of complete RPMI-1640 medium and incubated for additional 48 h. After incubation, a final concentration of 0.5 mg/mL MTT was added to the medium and then incubated at 37 °C for 1 h. The medium was removed and the dark blue formazan crystals dissolved in DMSO were determined spectrophotometrically at 535 nm by the multi-plate reader Genios Pro (Tecan, Austria) with Magellan 6 software, and the results are expressed as percentage of cell growth with respect to the control (untreated cells).

### 4.6. Synergy Analysis

The nature of the combination between [D-Gln^4^]LR-PEG liposomes and drugs (namely cDDP or RTX), combined at a fixed ratio either simultaneously or sequentially, was quantified by synergism quotient (SQ) [65,66]. SQ was defined as the net growth inhibitory effect of the analogue combination divided by the sum of the net individual analogue effects on growth inhibition. A quotient of > 1 indicates a synergistic effect, between 0.9 and 1.1 indicates an additive effect, while a quotient of < 1 indicates an antagonistic effect.

C13*, 2008 and IGROV-1 cell lines were exposed to the combinations using 3 different modalities. Simultaneous drug-exposure (sequence I): cDDP or RTX were given simultaneously with liposomes for 3 days; liposomes before (sequence II): [D-Gln^4^]LR loaded liposomes was given 24 h before cDDP or RTX; drugs before: the drug (cDDP or RTX) was given 24 h before liposomes (sequence III). 

All the incubation experiments were carried out according to the optimized protocol in which cells were exposed to liposome treatments for 15 h after the medium had been replaced with fresh one. Growth inhibition was assayed by MTT assay and the cytotoxicity of each combination was compared with the cytotoxicities of the drugs alone. Each experiment was performed at least 3 times. 

The heatmap and clustering have been realized with the open source software R and Bioconductor repository, using ggplot2 and Heatplus packages (https://cran.r-project.org/ [67]; https://www.bioconductor.org/ [68]). For the clustering (Euclidean distance, complete linkage clustering), in order to highlight the distance between antagonism, addition and synergy values, the synergism quotient values were elaborated as follow: for synergism quotient values < 0.9, a value of 10 was subtracted; for synergism quotient values ≥ 1.1, a value of 10 was added. 

### 4.7. Flow Cytometric Analysis of Cell Cycle

Quantitative measurements of the cell cycle phase distribution were performed by flow cytometry [69]. Cells were suspended in 0.5 mL of hypotonic fluorochrome solution (25 µg/mL PI, 0.1% sodium citrate, 0.1% Triton X-100). The samples were kept at 4 °C in the dark for at least 30 min, dispersed by repeated pipetting before flow cytometry analysis in a FACSCoulter Attune^®^ NxT Acoustic Focusing Cytometer (Invitrogen, Milan, Italy) equipped with a single 488 nm argon laser. The percentage of nuclei in the different phases of the cell cycle (G0/G1, S and G2/M) was calculated with a DNA cell cycle analysis software (Attune^®^ NxT Software version 1.0). A minimum of 1 × 10^4^ cells/sample were analysed for each sample.

### 4.8. Statistical Analysis

A statistical analysis was performed using the one-way analysis of variance (ANOVA). The data are represented as means ± SD. A difference was considered statistically significant at * *p* < 0.05, ** *p* < 0.01 or *** *p* < 0.005.

## 5. Conclusions

This work has demonstrated that the TS allosteric inhibitor, the [D-Gln^4^]LR peptide, showed a schedule-dependent synergistic antiproliferative effect against OC cells in combination with cDDP, RTX, 5-FU and paclitaxel. More precisely, simultaneous exposure as well as 24 h pre-treatment with the peptide, produced a synergistic cell killing, while a reversed schedule that consists in pre-treating with the non-peptidic drugs was always antagonistic. Furthermore, for the first time, we have provided evidence that this peptide can overcome resistance to cDDP, as its combination with this alkylating agent was synergistic also against the cDDP-resistant cell lines. Also, we have demonstrated a synergistic antiproliferative effect of the peptide in combination with 5-FU, or with RTX even against the C13* and A2780/CP cell lines that had been selected for their resistance to cDDP and cross-resistance to 5-FU and RTX, and feature amplified expression of the folate cycle enzymes [43]. Most of these effects paralleled with great alteration of cell cycle and additive or synergistic accumulation of apoptotic cells. Additionally, the present study indicates how liposome PEGylation, without changing the behaviour of the encapsulated drug, preserves its effectiveness and its therapeutic effect, making it comparable with that obtained with a specific peptide delivery system. Also, delivery by PEGylated liposomes allowed the carried peptide to be of effective formulations with other chemotherapy drugs. Notably, our results, in line with previous published evidence, show that, for a successful, synergistic functional combinatorial targeted therapy, careful attention must be paid to exposure, dose, schedule and target engagement. Thus, the results of this drug combination investigation concerning both in cytotoxicity and cell cycle studies imply the occurrence of complex biochemical interconnections between the peptide and the classical drugs used in this study. Many of these interactions involve apoptotic mechanisms accounting for the effects observed and discussed. 

Overall, the results of this preclinical study suggest that the association of standard drugs with this novel peptidic hTS inhibitor encapsulated into PEGylated pH-sensitive liposomes is a promising strategy to impact the drug resistance problem and, combined with intracellular mechanistic analysis, may represent a rational basis for future clinical applications in ovarian tumor patients.

## Figures and Tables

**Table 1:** Physicochemical characterization of the optimized unloaded and [D-Gln4]LR-loaded liposomes. Each value represents the mean ± SD; PDI: polydispersity index; DL: drug loading; EE: encapsulation efficiency.

Sample	Z-Average (nm)	PDI	Z-Potential (mV)	DL (µg/mg)	EE (%)
DOPE:CHEMS:DSPE_PEG (PpHL)	198 ± 30	0.279 ± 0.077	−14.54 ± 2.41	-	-
[D-Gln^4^]LR_ DOPE:CHEMS:DSPE_PEG ([D-Gln^4^]LR-PpHL)	209 ± 19	0.289 ± 0.072	−14.28 ± 2.19	21.03 ± 1.66	42 ± 3

## Supplementary Materials

Supplementary materials can be found at https://www.mdpi.com/1422-0067/21/12/4452/s1. Table S1. IC50 values (µM or nM) for selected compounds obtained after 72 h treatment of 2008, C13*, A2780, A2780/CP, and IGROV1 human ovarian cancer cell lines. Table S2. The effect of 72h-exposure to [DGln4]LR and 5-FU alone and in combination on the cell cycle phase distribution of 2008 and C13* cell lines by cytofluorimetric analysis. Table S3. The effect of 48h- and 72h-exposure to [DGln4]LR and cDDP alone and in combination on the cell cycle phase distribution of 2008 and C13* cell lines by cytofluorimetric analysis. Figure S1. Cell cycle-related analysis of the 2008 cells after treatment with the indicated compounds. Figure S2. Cell cycle-related analysis of the C13* cells after treatment with the indicated compounds. Figure S3. The effect of [DGln4]LR and cDDP alone and in combination on the cell cycle phase distribution of 2008 and C13* cells by cytofluorimetric analysis of the DNA content by PI staining.
