# Supplementary material for: A Peptidic Thymidylate-Synthase Inhibitor Loaded on Pegylated Liposomes Enhances the Antitumour Effect of Chemotherapy Drugs in Human Ovarian Cancer Cells
Source: Int J Mol Sci. 2020 Jun 23;21(12):4452. doi: 10.3390/ijms21124452 (PMC7352236; doi:10.3390/ijms21124452)
Supplement: Supplementary file 1 [file ijms-21-04452-s001.pdf]

International Journal of *Molecular Sciences*

## Supplementary Materials

**Table S1.** IC50 values (μM or nM) for selected compounds obtained after 72 h treatment of 2008, C13*, A2780, A2780/CP, and IGROV1 human ovarian cancer cell lines. Data indicate mean values and standard deviation from at least three experiments performed in duplicate.

| Compounds | 2008 cells | C13* cells | A2780 cells | A2780/CP cells | IGROV1 cells |
|---|---|---|---|---|---|
| **5-FU** | 6.4±0.7 | 9.3±1 | 8.5±0.8 | 11.6±1.2 | 16.9±1 |
| **[DGln4]LR** | 6.2±0.3 | 9.1±0.8 | 8.7±0.6 | 10.2±0.5 | 5.2±0.4 |
| **cDDP** | 3.4±0.1 | 15.5±1.3 | 5.2±0.6 | 20.6±1.5 | 9.8±1.1 |
| **RTX (nM)** | 27.7±2.3 | 42.4±3.8 | 16.2±1.2 | 28.3±4.5 | 21.2±1.6 |
| **Pacli (nM)** | 37.4±4.6 | 52.2±8.3 | 9.2±0.8 | 19.4±1.2 | 22.5±3.3 |

**Table S2.** The effect of 72h-exposure to [DGln4]LR and 5-FU alone and in combination on the cell cycle phase distribution of 2008 and C13* cell lines by cytofluorimetric analysis. The values are the mean of two experiments.

| | 2008 cells | | | | C13* cells | | | |
|---|---|---|---|---|---|---|---|---|
| | $G_0G_1$ | S | $G_2$/M | hypodiploid | $G_0G_1$ | S | $G_2$/M | hypodiploid |
| **Ctrl** | 75.19 | 9.78 | 10.83 | 1.25 | 67.58 | 11.71 | 11.62 | 0.37 |
| **[DGln4]LR** | 73.75 | 7.22 | 11.73 | 3.51 | 68.81 | 15.26 | 11.88 | 2.16 |
| **5FU 5-10uM** | 56.53 | 16.71 | 12.96 | 10.33 | 72.97 | 10.13 | 10.98 | 1.57 |
| **5FU 10-20uM** | 48.68 | 19.33 | 12.33 | 11.08 | 69.28 | 9.68 | 9.96 | 4.66 |
| **[DGln4]LR/ 5FU 5-10uM** | 53.22 | 13.93 | 8.05 | 19.07 | 69.62 | 11.05 | 11.33 | 2.78 |
| **[DGln4]LR/ 5FU 10-20uM** | 43.46 | 13.33 | 13.40 | 19.41 | 59.88 | 10.81 | 10.08 | 10.51 |

24 h after seeding, the cells were exposed to the drugs for 72h, then DNA content of untreated and treated cells was determined by flow cytometry after propidium iodide staining. Apoptotic cells are characterized by a lower DNA content (hypodiploid cells, having fewer than the diploid number of chromosomes) because of nuclear fragmentation.

**Table 3.** The effect of 48h- and 72h-exposure to [DGln4]LR and cDDP alone and in combination on the cell cycle phase distribution of 2008 and C13* cell lines by cytofluorimetric analysis. The values are the mean of two/three experiments.

| | **2008 cells** | | | | **C13* cells** | | | |
|---|---|---|---|---|---|---|---|---|
| **48 hr** | **$G_0G_1$** | **S** | **$G_2$/M** | **hypodiploid** | **$G_0G_1$** | **S** | **$G_2$/M** | **Hypodiploid** |
| **Ctrl** | 70.56 | 6.70 | 11.34 | 8.80 | 74.45 | 7.04 | 13.84 | 1.62 |
| **[DGln4]LR** | 61.18 | 6.95 | 7.60 | 20.55 | 74.77 | 9.25 | 10.77 | 1.69 |
| **cDDP** | 31.22 | 10.19 | 4.17 | 47.78 | 46.93 | 17.10 | 20.96 | 10.36 |
| **RTX** | 24.98 | 5.51 | 2.19 | 63.21 | 43.01 | 9.67 | 8.18 | 32.68 |
| **[DGln4]LR/ cDDP** | 23.18 | 9.67 | 2.87 | 58.72 | 29.52 | 15.31 | 30.54 | 20.33 |
| **[DGln4]LR/ RTX** | 22.46 | 6.90 | 3.13 | 63.13 | 30.66 | 9.61 | 7.18 | 50.01 |
| | **2008 cells** | | | | **C13* cells** | | | |
| **72 hr** | **$G_0G_1$** | **S** | **$G_2$/M** | **hypodiploid** | **$G_0G_1$** | **S** | **$G_2$/M** | **hypodiploid** |
| **Ctrl** | 62.90 | 9.96 | 14.37 | 2.56 | 62.61 | 13.30 | 11.87 | 8.72 |
| **[DGln4]LR** | 65.01 | 9.75 | 11.18 | 3.01 | 56.94 | 9.80 | 8.14 | 7.01 |
| **cDDP** | 31.24 | 8.82 | 9.61 | 41.87 | 17.73 | 12.18 | 45.83 | 18.44 |
| **RTX** | 29.51 | 4.33 | 6.77 | 55.61 | 33.05 | 8.65 | 11.24 | 36.41 |
| **[DGln4]LR/ cDDP** | 30.63 | 4.26 | 5.71 | 55.12 | 13.61 | 10.55 | 30.44 | 43.20 |
| **[DGln4]LR/ RTX** | 24.05 | 2.54 | 4.51 | 66.61 | 37.87 | 6.34 | 6.66 | 48.98 |

24 h after seeding, the cells were exposed to the drugs or 48h or 72h, then DNA content of untreated and treated cells was determined by flow cytometry after propidium iodide staining. Apoptotic cells are characterized by a lower DNA content (hypodiploid cells, having fewer than the diploid number of chromosomes) because of nuclear fragmentation.

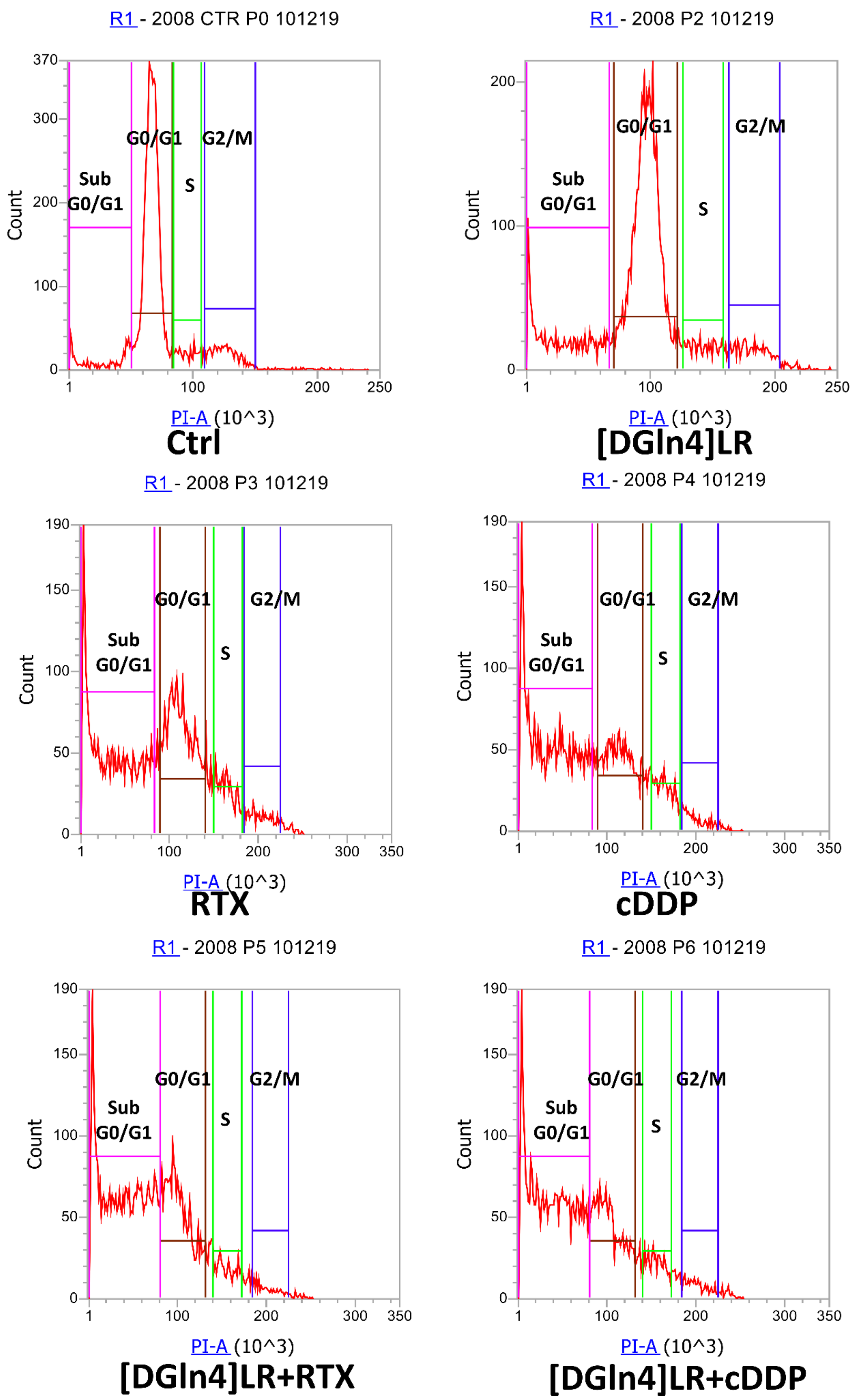


**Figure S1.** Cell cycle-related analysis of the 2008 cells after treatment with the indicated compounds. The variations of the cell distribution in the different phases of the cell cycle and especially in the sub-G0G1 area after the various treatments are shown. The results are representative of two/three independent assays.

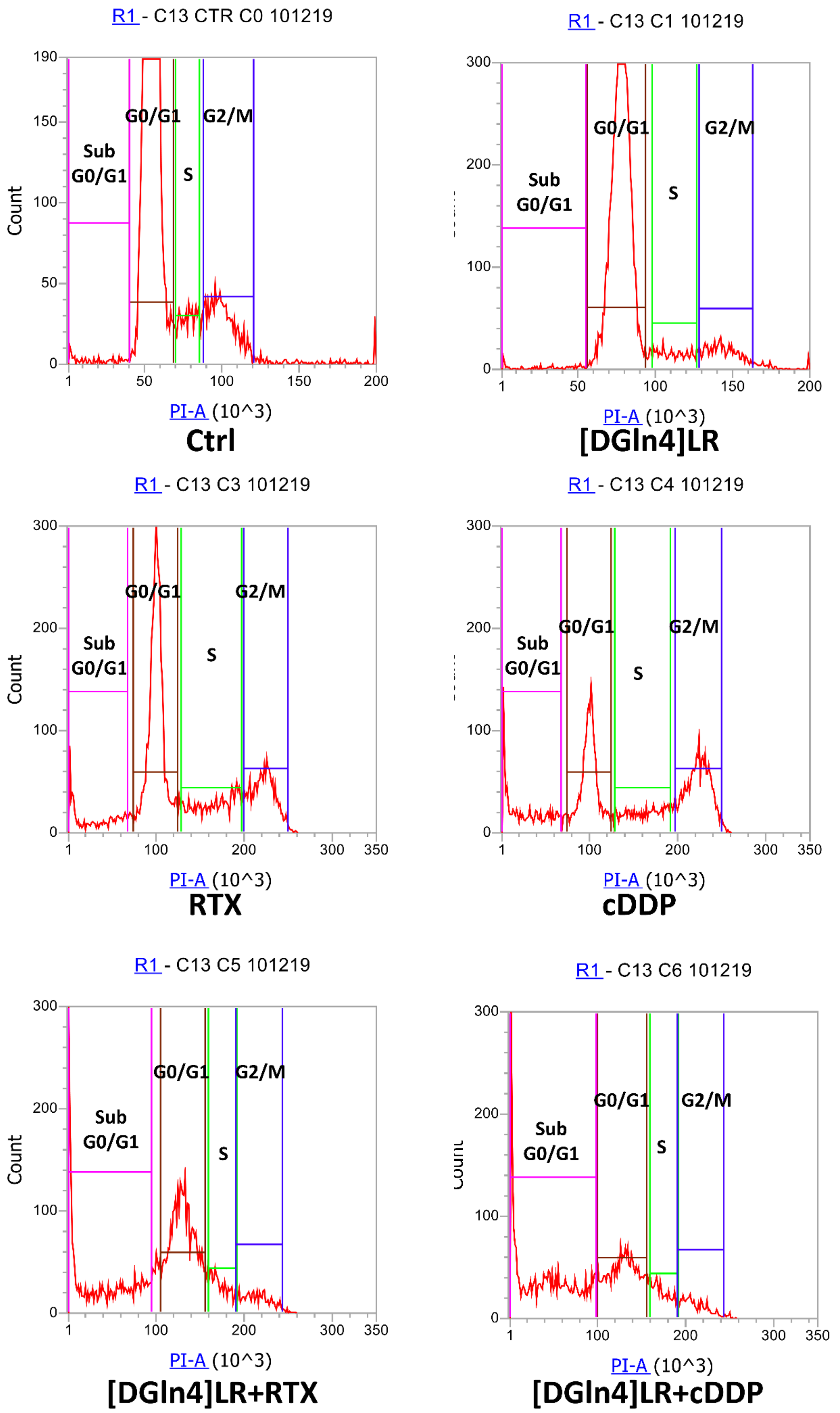


**Figure S2.** Cell cycle-related analysis of the C13* cells after treatment with the indicated compounds. The variations of the cell distribution in the different phases of the cell cycle and especially in the sub-G0G1 area after the various treatments are shown. The results are representative of two/three independent assays.

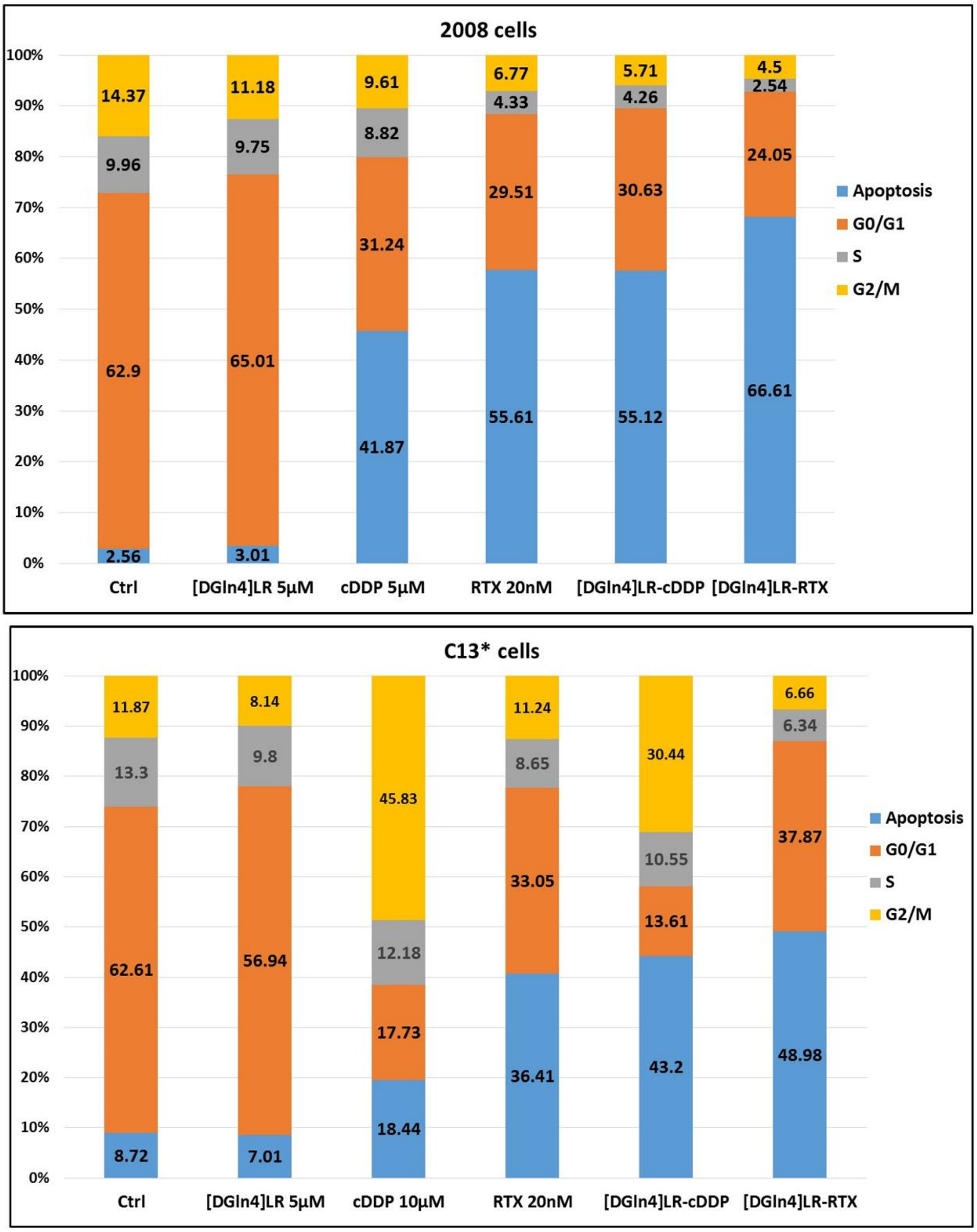


**Figure S3.** The effect of [DGln[4]]LR and cDDP alone and in combination on the cell cycle phase distribution of 2008 and C13* cells by cytofluorimetric analysis of the DNA content by PI staining. After 48-exposure to 5 µM [DGln[4]]LR and 5µM (2008 cells) or 10µM (C13* cells) cDDP, RTX 20nM alone and in concurrent combinations, cells were processed according to materials and methods. Inserted numbers indicate the % of cells in the different phases of the cell cycle. The values are the mean of two/three experiments. The error bars are omitted for a clearer visualization.
